# Underground Inter-Nest Tunnels of Red Imported Fire Ants, *Solenopsis invicta*: Physical Features and Associations with Colony and Environmental Factors

**DOI:** 10.3390/insects16080835

**Published:** 2025-08-13

**Authors:** Meihong Ni, Juli Lu, Xinyi Yang, Yiran Zheng, Yuan Wang, Mingxing Jiang

**Affiliations:** Ministry of Agriculture and Rural Affairs Key Laboratory of Molecular Biology of Crop Pathogens and Insect Pests, Zhejiang Key Laboratory of Biology and Ecological Regulation of Crop Pathogens and Insects, Zhejiang Engineering Research Center for Biological Control of Crop Pathogens and Insect Pests, Institute of Insect Sciences, Zhejiang University, Hangzhou 310058, China; ni_meihong@zju.edu.cn (M.N.); 22316094@zju.edu.cn (J.L.); xyyang1026@163.com (X.Y.); yiranzheng@zju.edu.cn (Y.Z.); 22016105@zju.edu.cn (Y.W.)

**Keywords:** *Solenopsis invicta*, tunnel system, nest relocation, social form

## Abstract

While foraging tunnels of the red imported fire ant, *Solenopsis invicta*, have been well studied, much less is known about the tunnels constructed between neighboring nests, despite their perceived importance in intra-colony exchange and collaboration. In this study, we investigated such tunnels by excavating 80 pairs of nests (with distances of <1 m between nests) located in different types of habitats. For each pair of nests, we recorded the number of inter-nest tunnels and observed their shape, diameter, subsurface depth, and ant presence within them. Moreover, we analyzed the relationships between the probability of constructing inter-nest tunnels and several nest/habitat characteristics, including distance between nests, colony social form, nest size, soil type, and vegetation cover, as well as the relationships between tunnel numbers and these factors. The results show that the number of inter-nest tunnels ranges from one to 11. These tunnels open to the two nests at terminals, are elliptical in cross-section, <1.5 cm in diameter, and mostly at 1–3 cm (range 1–12 cm) subsurface depth. Among the 36 pairs of nests possessing tunnels, 31 pairs (86.1%) had worker or alate ants within their tunnels. Polygynous colonies are more likely (52.4%) to construct inter-nest tunnels than monogynous colonies (17.6%). Nest pairs that have a small nest, located in habitats with higher vegetation cover and loamy or sandy loam soil, tend to have inter-nest tunnels. We also showed that the capacity of constructing inter-nest tunnels falls in the regime similar to foraging tunnels. As nests were treated with chemicals, 33 nests were relocated and 47 new nests resulted within 2 weeks, but no definite tunnels were constructed between original nests and corresponding new nests. Our results highlight the significance of including such tunnels when analyzing intra-colony exchange, collaboration, and adaptive strategies in *S. invicta*. Uses of tunnels by fire ants during nest relocation, and the requirement of destroying them during control program implementation, were discussed.

## Simple Summary

Red imported fire ants (*Solenopsis invicta*) are well known for their elaborate foraging tunnel system; however, little is known about the tunnels constructed between neighboring nests (inter-nest tunnels) which may serve important functions in colony communication and cooperation. In this study, we investigated 80 pairs of neighboring nests across various habitats, and found that 45% of them were connected by 1–11 inter-nest tunnels. These tunnels exhibit several distinctive characteristics: they terminate at both nests of a pair, display elliptical cross-sections (<1.5 cm in diameter), typically run just centimeters below the surface, and frequently contain worker or alate ants. Notably, polygynous colonies (containing ≥2 queens) are more likely (ca. 52.4%) to construct inter-nest tunnels compared to monogynous colonies (approximately one-third as frequent). Moreover, if the nests are small, located in habitats with high vegetation cover and loamy or sandy loam soil, the inter-nest tunnels are more likely to be constructed. We also evaluated preliminarily fire ants’ capacity for digging such tunnels, and their probability of being induced to dig such tunnels when colonies face external threats (e.g., insecticide exposure). These findings highlight the ecological significance of inter-nest tunnels in *S. invicta* colonies, and we recommend increased research attention to these tunnels, both for understanding fire ant biology and developing more effective control strategies.

## 1. Introduction

Nests of ants serve as central places for rearing their broods and maintaining colonies. For ground-nesting ants, nests typically consist of a subterranean network of shafts, chambers, and tunnels, and they may have an above-ground mound in some species [1]. Around the mound, there are foraging tunnels that often radiate outward, connecting the mound to various foraging sites [2,3]. In addition to extending the foraging range, foraging tunnels help ants protect food during transportation and shield them from predators, extreme temperatures, and desiccation [4,5]. In polydomous ant colonies (i.e., the colonies living in at least two physically separated but socially connected nests), foraging tunnels may link two or more nearby nests [6], creating a network that benefits colony life by spreading risk between nests, optimizing foraging, and mitigating the effects of nest-level constraints [7]. For example, when facing insecticides applied to nests, ants may use foraging tunnels as an approach to move to nearby nest(s) or other safe sites [6]. Therefore, studying ant tunnels can not only offer valuable insights into their adaptive strategies, but also help explain the low potential efficacy of control measures [7,8,9]. So far, most research on such tunnel-linked nest connections has centered on polydomous colonies and their intra-colony sharing [7]. Meanwhile, given that tunnel networks often overlap in dense ant populations, inter-colony sharing via these tunnels is also possible.

The red imported fire ant, *Solenopsis invicta* Buren, native to South America, is one of the most destructive, invasive ground-nesting ants worldwide [10]. It has spread to the United States, the Caribbean, Oceania, Eastern Asia, and recently Europe [11,12], causing serious economic losses and biodiversity reduction in some regions [13,14,15]. *S. invicta* has two social forms, polygyne and monogyne, with its polygyne form typically occurring as polydomous colonies [16]. Foraging tunnels of *S. invicta* have long been a research interest, and their distribution and features are now well understood [2,17,18,19]. However, despite the knowledge that foraging tunnels (and also possibly other types of tunnels) contribute a lot to the inter-nest connections [6], little is known about how they are constructed between neighboring nests (particularly those of polydomous colonies), a key aspect to be clarified when evaluating their roles.

Previous research has shown that factors such as social form (polygynous versus monogynous), soil type and vegetation density influence the nest structure of ants. For instance, polygynous *S. invicta* colonies, which have multiple queens, tend to form inter-nest connections more readily because neighboring nests exhibit reduced worker aggression [6,16]. Lower bulk density of clay soils would better facilitate construction of foraging tunnels and allow for deeper colony expansion; a larger particle size in sandy soils and less clay for particle bridging can reduce soil strength, resulting in fewer foraging tunnels [20]. Leaf litter, which may affect ants’ movement on the ground [21,22], possibly affects ants’ decision of whether to dig tunnels for convenient underground transportation. Mature colonies often prefer nesting sites with taller vegetation, because such sites are less disturbed and the vegetation provides support and structure as the mounds increase in size [23,24]. So far, it is unclear whether inter-nest tunnels are also related to some of these factors. Studying these associations can depict the conditions favorable for constructing such tunnels.

Whether the construction of inter-nest tunnels is possibly induced by control measures is an interesting question. We address this because, during the period following insecticide use, nest relocation by *S. invicta*, i.e., ants leaving the original nest and building a new one, can be frequently observed (e.g., [25,26,27]), and we suppose that tunnels might be constructed between these two nests. If tunnels have existed between nests, then ants may use these tunnels to leave endangered nests quickly for the connected nests or temporarily stay within these tunnels. In each of these cases, the control efficacy may be reduced. Therefore, in terms of *S. invicta* monitoring and control requirements, inter-nest tunnels are also of research significance.

In this study, we investigated 80 pairs of *S. invicta* nests, recording the presence and number of inter-nest tunnels, mound size, social form, and environmental factors, including soil type, vegetation cover, and leaf litter, and analyzed their associations. Additionally, we measured the length of foraging tunnels from 58 solitary nests to compare with the length of inter-nest tunnels, in order to provide insight into the capacity of *S. invicta* to construct inter-nest tunnels. Moreover, we observed the relocation of nests as a response to insecticide use, the number of tunnels extending from the original nest to the new ones, and ants present in these tunnels. Our aim was to create an overall profile of these tunnels, identify key factors that influence them, and contribute to a better understanding of the adaptive significance of *S. invicta*’s underground network.

## 2. Materials and Methods

### 2.1. Locations, Habitats, and Nest Mounds

The investigation was conducted at four locations (Wucheng, Jindong, Dongyang, and Longyou) in Zhejiang Province, Eastern China, from July 2020 to May 2025. These locations encompass various local habitats favored by *S. invicta*, including green belts, grasslands, roadsides, and ridges of farmlands. In green belts, the dominant plants around nests were *Cynodon dactylon* (L.) Pers. and/or *Zoysia matrella* (L.) Merr., while in the other habitats they were annual and perennial grasses, primarily consisting of species from *Digitaria*, *Setaria*, *Eragrostis*, or *Paspalum*, with a sparse presence of forbs such as *Alternanthera philoxeroides* (Mart.) Griseb., *Erigeron annuus* (L.) Pers., *Sonchus oleraceus* L., and shrubs like *Rubus hirsutus* Thunb.

### 2.2. Nest Mounds

In each habitat, we first located all potential mounds meeting our spatial criteria as stated below through systematic surveying. From these identified mounds, we then randomly selected the planned number of mounds, using a computer-generated random number sequence (Microsoft Excel’s RAND() function) applied to a list of the qualifying mounds. As a result, the mounds used in our study were randomly selected, located in diverse sites that differed in vegetation height, vegetation density, soil characteristics, and litter depth. This diversity facilitated analysis of how these factors correlate with tunnel formation.

We investigated two types of mounds: mounds of 160 “paired nests” (i.e., 80 pairs) and mounds of 58 solitary nests (Table 1). The two mounds of paired nests were generally of different sizes, less than one meter apart, for studying inter-nest tunnels. We did not know whether the two paired nests belonged to the same colony or two colonies, yet both possibilities exist because nests very close to each other (e.g., separated by <1 m) may belong to different colonies [28]. The mounds of solitary nests were situated at least 2 m from the nearest mound nests and were randomly selected from the sites for paired-mound investigations. The solitary mounds were used to establish a baseline for foraging tunnel lengths, which were then compared with inter-nest tunnel lengths to assess whether the latter followed a characteristic pattern, such as matching the length of foraging tunnels.

One week before the investigation, the length, width, and height of each mound, along with the distance between them (for paired mounds), were measured to the nearest 0.5 cm. Then, each selected mound was chemically treated to eliminate the ant colonies. The treatment consisted of two steps: first, we applied Kai Rui^®^ baits (0.73% hydramethylnon, BASF, Ludwigshafen, Germany) around each mound at a dose of 20 g per mound; second, after 3–4 days, we poured a mixture of 5% imidacloprid and 40% chlorpyrifos into the nests. The margins and surrounding areas of the treated mound were left intact, as required for tunnel excavation. Worker ants were collected from each mound, and the social form of the colony was determined using the methods of Valles and Porter [29].

### 2.3. Excavation of Mounds and Recording of Habitat Features

#### 2.3.1. Paired Mounds

Before excavation, we documented the habitat types for each selected paired mound (80 pairs in total), classifying the habitats into four categories as mentioned previously: green belt, grassland, roadside, and farmland. For each habitat, we recorded the predominant vegetation within a 1 m radius around the mounds, noting the plant status, density, and height of the dominant species. Overall vegetation density was assessed based on ground coverage, classified into three levels: 0 (bare), 1 (vegetation cover <50%), and 2 (vegetation cover ≥50%).

Grasses between the two mounds of the selected pair were trimmed as closely as possible. Then, a trench, ca. 20 cm deep, 10–15 cm wide, and 50–70 cm long, was excavated with a small spade and forceps approximately midway between the two mounds to create a cross-section. We carefully examined the cross-section for round or elliptical holes (indicative of tunnel interruptions or foraging trails) and checked for the presence of ants within these holes. If holes were found, they were marked with flags, and the vertical distance from each hole to the ground was measured to the nearest 1 mm. We typically waited at least 20 s for residual ants (those surviving after control measures) to exit the intersection. If no holes were observed in the cross-section, we used forceps to scrape the soil in search of holes that might have been obscured during excavation. If no tunnel was confirmed, we ceased excavation at that site. Excavation continued along the tunnel toward the mounds until reaching them; during this process, marking flags were used to locate tunnel positions as needed. Special attention was given to tunnels that might have collapsed prior to excavation and could be easily missed during investigation; such tunnels typically leave distinct residues characterized by rounded forms and smooth wall fragments, making them readily identifiable. This careful examination is especially critical in sandy soil conditions, where the larger particle size in soils and less clay for particle bridging would reduce the strength of tunnels [20].

Throughout the excavation, we noted the soil conditions around the mounds and classified them into two types: (a) soil dominated by loam, sandy loam, or shallow loam with underlying clay; and (b) soil primarily composed of sand or gravel, with minimal loam.

#### 2.3.2. Solitary Mounds

To determine the foraging tunnel length of solitary nests, which, as stated previously, were essential for assessing inter-nest tunnel length, we excavated the foraging tunnels of each selected solitary nest (58 in total). Habitat type, vegetation cover, and soil type were recorded for each selected mound, as for the paired mounds.

For each mound, we aimed to identify its longest foraging tunnel, defined as the tunnel extending from the mound’s edge to the furthest exit. This was achieved by excavating trenches around the mound in concentric circles at distances of 20, 40, and 60 cm from the mound’s edge, starting with the 60 cm circle. Each trench was dug to a maximum depth of 20 cm, which, as Markin et al. [2] noted, is sufficient to expose subterranean tunnels radiating from the mound.

If tunnels were discovered at the 60 cm transect, we excavated them sequentially toward the mound. If a tunnel ended with an exit (before reaching the mound’s edge), we ceased excavation of that tunnel and shifted to the next one. Once a tunnel reaching the mound’s edge was located, we continued digging it in the reverse direction until we reached the nearest exit. In this manner, we excavated all tunnels present at the 60 cm transect and selected the longest one for further analysis. If no tunnels were found at the 60 cm transect, we then excavated trenches along the 40 cm concentric circle and investigated the tunnels as described above.

### 2.4. Relations of Tunnels to Nest Relocation

To investigate whether tunnels are used as a pathway by ants during nest relocation, we treated nests with insecticides to induce ants to construct a new nest nearby, and then examined the number of tunnels situated between the treated mound and the mound of the new nest, using the method described above for investigating inter-nest tunnels. This study was conducted from late April to early May of 2025, in Jindong of Zhejiang Province, at a site (ca. 1.0 ha.) with habitats favorable for *S. invicta*, like orchards, grasslands, nurseries, and field ridges. Ninety-five *S. invicta* mounds (>20 cm in diameter) were selected randomly using the same method as described in Section 2.2, i.e., via Microsoft Excel’s RAND() function, without considering their locations and habitat types. They were first applied with Kai Rui^®^ toxin baits as described above, then at 1, 2, 7, and 14 days after the treatment, the number of new mound(s) formed within a range of 5 m from treated mounds was counted. The new mounds were defined as those >5 cm in diameter, and at least 20 worker ants would appear on the mound within 1 min after disturbing the mound. On day 14, we measured the distance between new mounds and their corresponding original mounds, dug the soil between them to observe inter-nest tunnels, using the method as described above.

### 2.5. Statistical Analyses

The approximate size of the nests was calculated based on the size of the mounds. For paired nest datasets, Chi-square tests were performed to measure associations between connectivity (whether or not the nests were connected) and the factors social form, soil type, and vegetation state. To evaluate the significance of inter-nest distance and nest size, we employed a *t*-test (for normally distributed data with equal variance) or a Mann–Whitney *U* test (for non-normally distributed data); for nest size, we performed analysis separately (1) within the nests that comparatively had a bigger size (size-LN) in the pair, (2) within the nests with a smaller size (size-SN), and (3) within the average size of the two nests (size-Avg).

For the number of inter-nest tunnels, Spearman’s Rho tests were performed to assess their correlations with various factors, i.e., inter-nest distance, size-LN, size-SN, and size-Avg, and *t*-tests were performed to evaluate the significance of vegetation cover (<50% versus ≥50%, excluding the “bare” group from analysis, which had only two cases). No tests were performed for correlations with social form and soil type, because there were not sufficient cases that could be used for analysis.

For solitary nest datasets, similar analyses were performed to examine the relationships between the longest foraging tunnels and nest size, soil type, and vegetation cover. SPSS 25.0 [30] and GraphPad Prism 10.0 [31] were employed for analyses.

## 3. Results

### 3.1. Features of the Tunnels Between Adjacent S. invicta Nests

To uncover the tunnels between neighboring *S. invicta* nests, we sampled 80 pairs of mounds (i.e., 160 mounds) and excavated the soil between the two mounds for each pair (Table 1). Among these pairs, 45% (36 pairs) contained inter-nest tunnels (hereafter referred to as “connecting tunnels”), which opened into both nests. Additionally, we frequently encountered tunnels that had exits to the ground surface and did not connect to the other nest in the pair. Some pairs exhibited small soil piles (3–5 cm in diameter) on the surface, likely transported underground by ants during tunnel excavation. Both worker and alate ants were present in the connecting tunnels, accounting for 86.1% (31/36) and 77.8% (28/36), respectively, of the pairs with such tunnels. A few pairs (3/36) contained only worker ants, while the remaining pairs (5/36) lacked ants of either kind.

**Figure 1:**
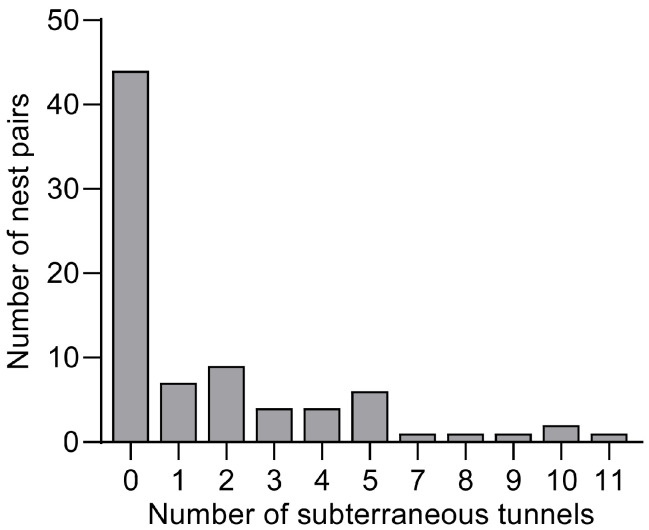
Number of nest pairs exhibiting different numbers of subterraneous inter-nest tunnels (n = 80 pairs).

Each pair had one to eleven connecting tunnels (Figure 1). The tunnels typically had a cross-sectional height of around 1 cm, with varying shapes and sizes, sometimes exhibiting significant variations as they extended through the soil. Some tunnels took sudden turns when encountering gravel or stones. The vertical distance of the tunnels to the ground surface varied, generally ranging from 1 to 3 cm. However, some tunnels were very close to the surface, while others extended to greater depths of 8 to 12 cm. When multiple tunnels were present, they were largely parallel to one another and occasionally connected at certain points.

**Figure 2:**
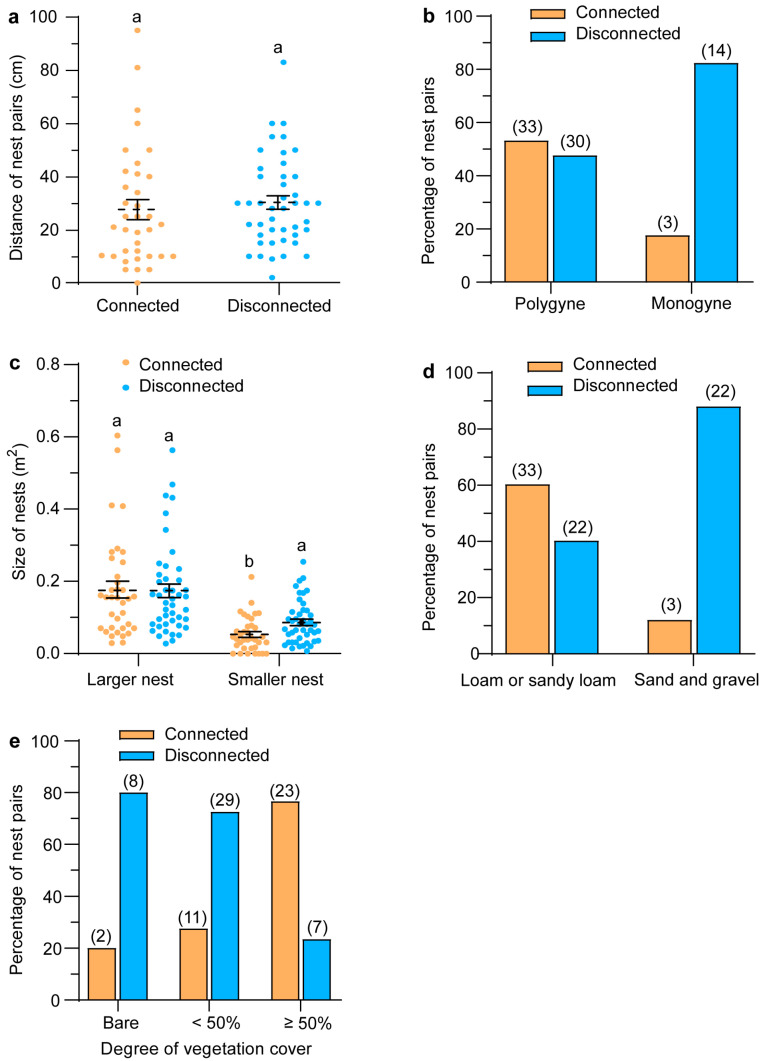
Relationships of *S. invicta* inter-nest connectivity state with inter-nest distance (**a**), colony social form (**b**), nest size (**c**), soil type (**d**), and vegetation (**e**). Arabic numerals above columns indicate the corresponding number of grouped nest pairs. Data in panels (**a**,**c**) are presented as mean ± SE. Bars with the same letters indicate no significant difference (*p* > 0.05, Mann–Whitney *U* or *t*-tests).

When inter-nest tunnels were present, the nests were typically 5 to 50 cm apart (median: 21.5 cm), with some distances reaching 80 to 100 cm. This distance was smaller than that of pairs without inter-nest tunnels, which ranged from 10 to 60 cm (median: 29 cm; Figure 2a), although the difference was not statistically significant (*Z* = −1.206, *p* = 0.228; Mann–Whitney *U* test).

### 3.2. Associations of Inter-Nest Tunnels with Environmental Factors and Social Form

The presence of inter-nest tunnels significantly correlated with social form (*p* = 0.011), nest size (*p* = 0.008 for the smaller nest), soil type (*p* < 0.001), and vegetation cover (*p* < 0.001; Chi-square or *t*-test), but not with nest distance as described above (Table 2). Nests were more likely to be connected by tunnels in polygynous colonies. Of the 80 investigated pairs, 63 pairs (nearly 80%) belonged to polygynous colonies, while the remaining (17 pairs) were monogynous. Among the polygynous pairs, over half (52.4%) had underground connecting tunnels (Figure 2b). In contrast, only 17.6% of the monogynous pairs had such tunnels, which is roughly one-third the rate observed in polygynous pairs.

Nests were more likely to be connected if one was significantly smaller, and if they were located in areas with abundant vegetation or dominated by loam or sandy-loam soils (Figure 2c–e). Some small nests, even those without an evident mound, could be connected by tunnels to neighboring nests 10 to 40 cm apart. Soils dominated by sand or gravel were less conducive to the formation of connecting tunnels. Conversely, the number of tunnels did not significantly correlate with any of the four analyzed factors: nest distance (*ρ* = 0.068, *p* = 0.707; Spearman’s Rho test), nest size (size-LN: *ρ* = 0.176, *p* = 0.328; size-LN: *ρ* = −0.069, *p* = 0.704; size-Avg: *ρ* = 0.206, *p* = 0.250; Spearman’s Rho test), and vegetation (*t* = −0.591, df = 32, *p* = 0.559, *t*-test).

Furthermore, inter-nest tunnels can be constructed regardless of the state of litter on the ground surface. Of the 14 nest pairs sampled in areas with litter (1–3 cm in depth), six pairs were found to have underground connecting tunnels. Notably, at one roadside with abundant vegetation and 2–3 cm of litter on the ground, we discovered two nests (45 cm apart) connected by a single nearly horizontal tunnel located approximately 4 cm deep. At another roadside with similar vegetation and litter conditions, we found one pair of nests (10 cm apart) connected by three tunnels at depths of 3–8 cm.

### 3.3. Comparisons of Inter-Nest Tunnels with the Foraging Tunnels

Of the 58 isolated nests, 27 nests (46.6%) contained foraging tunnels directly connected to the nest mounds. The length of the “longest” foraging tunnels (referred to as “foraging-tunnel length”) ranged from 3 to 90 cm (median: 19 cm), with most measuring between 10 and 40 cm; 7.4% exceeded 50 cm. Their average length (22.8 ± 3.4 cm) was 17.7% shorter than the length of inter-nest tunnels (27.7 ± 3.7 cm; = the distance between the two nests in pairs as shown in Figure 2a), although they did not differ significantly (*Z* = −0.633, *p* = 0.527, Mann–Whitney *U* test). Therefore, digging a tunnel from one nest to the other nearby appears to be not difficult for *S. invicta*, as can also be revealed by the number of inter-nest tunnels, which reaches up to 11, as previously described.

This finding suggests that *S. invicta* faces minimal difficulty in excavating tunnels between neighboring nests, a conclusion further supported by the observation of up to 11 inter-nest tunnels connecting some nest pairs, as previously described.

**Figure 3:**
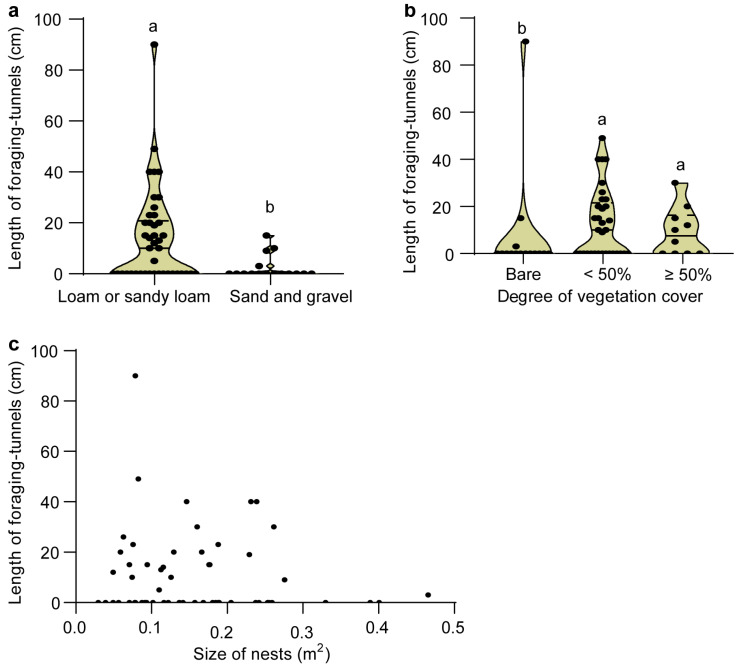
Relationships between the length of the longest foraging tunnels of *S. invicta* (n = 58) and soil type (**a**), vegetation (**b**), and nest size (**c**). Values with the same letters indicate no significant difference (*p* > 0.05, Mann–Whitney *U* test).

Foraging-tunnel length was significantly correlated with soil type (*ρ* = −0.339, *p* = 0.009, Spearman’s Rho test), with longer tunnels found in loam or sandy loam soils compared to those dominated by sand or gravel (Figure 3a). A close correlation was also observed between foraging-tunnel length and vegetation cover (*ρ* = 0.242, *p* = 0.068), with longer tunnels in areas with denser vegetation (Figure 3b). However, no significant correlation was found between foraging-tunnel length and nest size (*ρ* = −0.049, *p* = 0.714; Figure 3c). Thus, both inter-nest connecting tunnels and foraging tunnels in isolated nests are closely related to soil type and vegetation state, but less so to nest size, displaying a similarity in associations with environmental factors.

### 3.4. Contributions of Tunnels to Nest Relocation

Among the 95 nests investigated, 13, 9, 8, and 3 nests (33 nests in total) were observed to relocate at 1, 2, 7, and 14 d after the Kai Rui^®^ treatment, which accounted for 13.7%, 9.5%, 8.4%, and 3.2% (35% accumulatively), respectively. Most relocations took place within a week after treatment, particularly the first 2 d. By day 14, 47 new nests in total were produced as a result of relocating, of which only 18 nests survived to date, with each present with an evident mound (>5 cm in diameter) on the ground. Of these 18 nests, only one nest had a tunnel that extended 2.5 m, with openings to the ground, and reached its original nest at the terminus. However, it remains unclear whether this tunnel had been present before our observation or whether it was newly constructed. The ants in this tunnel and the two connected nests were very active during the 2 weeks after treatment.

## 4. Discussion

In this study, we demonstrated a kind of tunnel constructed by *S. invicta* that is situated between two neighboring nests. Our observations indicate that such tunneling behavior is prevalent in *S. invicta* colonies, with nearly half (45%) of nest pairs exhibiting interconnections, ranging from 1 to 11 tunnels per pair (Figure 1). These tunnels were typically shallow (1–3 cm belowground) and lacked surface openings; they differed distinctly from foraging tunnels, which are 3–10 cm deep and often have openings or exits to the ground [2]. Inter-nest tunneling occurred in both polygynous and monogynous colonies, with its frequency being higher in the former, and influenced by a few colony-specific and environmental factors as well. These findings contribute to a broader understanding of *S. invicta* tunnel networks and may aid in analyzing adaptive strategies in ant behavior.

Interestingly, the prevalence of inter-nest tunnels appears related to colony social structure, with polygynous colonies more likely to construct such tunnels. This is consistent with the well-documented behavioral differences between the two social forms: polygynous colonies are normally less aggressive toward each other [16], and their nests are more likely to take a polydomous form (i.e., the colony tends to live in multiple nests; [32]). The discovery of these tunnels provides a mechanistic explanation for the efficient exchange of workers and resources among nest units in polydomous systems, as previously reported [33,34].

Interestingly, our study also revealed that monogynous colonies can construct inter-nest tunnels when employing a polydomous nesting strategy, albeit at a lower frequency of 17.6% (3/17). As a result, monogynous colonies can maintain cooperative relationships between nests, facilitating resource exchange. This finding contrasts with the traditional view that workers from different nests of the same monogynous colony are typically hostile toward each other [16]. This is one of the few novel findings about tunnel systems of monogynous *S. invicta* colonies, which were previously reported by Cassill et al. [3].

Soil properties, including particle size, density, moisture retention capacity, and cohesiveness, can significantly influence ant tunneling behavior by affecting excavation efficiency and tunnel stability [3,20,35,36,37,38]. In this study, *S. invicta* colonies in gravel-dominated soils exhibited reduced inter-nest tunnel construction. This may be attributed to: (1) soil particles exceeding the ants’ carrying capacity, (2) decreased tunnel stability in coarse substrates. Additionally, interactions between soils and vegetation may also modulate tunneling patterns. For instance, clay soils typically support denser root systems than sandy soils [3], and thus fire ants may shift their excavation strategies along with changes in root density.

We found that vegetation cover can significantly influence inter-nest tunnel formation, with higher plant coverage promoting tunnel development. This could be explained by several aspects: (a) Dense vegetation tends to support richer food resources for ants (e.g., herbivorous arthropods), and underground tunnels may enhance the accessibility to these resources [39]. (b) Dense vegetation may also harbor more natural enemies; inter-nest tunnels would contribute to the coordination of defensive forces among nests. (c) Vegetation generally reduces solar radiation at ground level while increasing root density, which collectively improve soil moisture retention, a critical factor for tunnel stability [36]. Additional vegetative factors may also contribute, as vegetation cover [40,41,42] and vegetation height [43] can influence the abundance of worker ants, the tunnel builders. Interestingly, while ground litter (a vegetative byproduct) affects tunnel construction in other systems [21,22], we found no clear association in our study (tunnels occurred in only 6 of 14 nest pairs at sites with 1–3 cm litter). The possible reason is that leaf litter, especially if loose, might enable colonies to make protected inter-nest pathways without tunneling as the ants could travel between leaf layers. Efforts are needed to study other factors, such as temperature and colony plasticity, for their effects on tunnel construction [44].

Interestingly, smaller *S. invicta* nests tend to have a higher probability of forming inter-nest tunnels (Table 2, Figure 2c). Particularly, even the small nests that lack an obvious mound were connected by tunnels to larger nests. Yet, we did not know which nest (i.e., the bigger or smaller one) made the connection, though it is clear that polygynous colonies would have to make new smaller nests for expansion so as to develop denser populations [45]. We also do not know what significance the inter-nest tunnels had for the colony, e.g., serving as temporary secondary sites established to facilitate resource transportation and colony communication; products of budding behavior in polygynous colonies. Yet, we suppose that, by connecting to a larger nest nearby, inter-nest tunnels may help the ants in small nests better withstand attacks from natural enemies, and mitigate competitive pressures from native ants [46] or from other fire ant colonies [47]. More research is needed to clarify the functions of these tunnels connecting small nests. Further investigation is required to clarify this supposition.

As an energy-intensive process, tunnel construction requires careful resource allocation, as the invested energy could be used to improve colony performance [21]. In this context, tunnel length serves as a critical metric for evaluating resource expenditure. Our comparison revealed that inter-nest tunnels and the longest foraging tunnels exhibit similar lengths, suggesting that *S. invicta* constrains the energetic cost of digging inter-nest tunnels to levels comparable with foraging tunnels. Furthermore, the absence of a significant correlation between inter-nest tunnel length and the distance between neighboring nests (Figure 2a) implies that additional factors (e.g., nest size and vegetation cover, as previously discussed) likely play a more decisive role in determining whether such tunnels are constructed.

Our findings suggest that tunnels play a minimal role in the nest relocation of *S. invicta*. Of the 18 newly formed nests that survived for at least two weeks, only one maintained a tunnel connecting it to its original nest. In other words, fire ants are not ready to excavate new tunnels during relocation, presumably because ground movement is more energy-efficient than tunneling. Instead, inter-nest tunnels are likely to play roles in the colony’s other life processes, such as budding and post-budding expansion.

From a pest control perspective, disrupting these inter-nest tunnels, for example, by excavating soil around mounds, could effectively prevent ant movement between nests. Additionally, we recommend destroying inactive or abandoned nests near active ones, as they may serve as potential sites for colony re-establishment via residual tunnels.

## 5. Conclusions

Our study reveals that *S. invicta* commonly constructs inter-nest tunnels between neighboring nests, which likely serve important functions in colony dynamics while simultaneously reducing control measures efficacy. These shallow (1–3 cm depth) tunnels have significant associations with colony social structure (being more prevalent in polygynous colonies), soil types (loam/sandy-loam preferred), and vegetation cover, but exhibit no correlation with inter-nest distance. Notably, smaller nests showed higher tunnel connectivity (i.e., connected by tunnels to larger nests), suggesting adaptive benefits of inter-nest tunnels under ecological pressures. Importantly, our findings indicate that when chemically controlling fire ants, destroying tunnels cannot prevent colony relocation. As the first comprehensive characterization of inter-nest tunneling in fire ants, this work provides crucial insights into the environmental and social factors driving tunnel construction, advancing our understanding of fire ant subterranean networks. Future studies should investigate the timing of tunnel excavation and its specific functional roles in colony organization and survival. It is also important to determine whether the tunnel-connected nests belong to one single colony or two unrelated colonies.

## Figures and Tables

**Table 1:** Information on the 80 paired and 58 isolated nests of *Solenopsis invicta* sampled for the observation of inter-nest tunnels and foraging tunnels in Zhejiang Province, Eastern China.

Sampling Locations	Coordinates	Habitat Type	No. of Sampled Nests
Wucheng, Zhejiang	29.028~29.169° N, 119.592~119.738° E	Green belt and grassland	31 paired nests and 9 isolated nests
Dongyang, Zhejiang	29.095~29.116° N, 120.201~120.230° E	Roadside, grassland, and farmland	42 paired nests and 49 isolated nests
Longyou, Zhejiang	29.083~29.085° N, 119.264~119.265° E	Farmland	7 paired nests

**Table 2:** Analysis of associations between the occurrence (presence or absence) of subterraneous inter-nest tunnels and various factors.

Factors	Values Produced from Tests	*p*-Value	df
Nest distance	−1.206	0.228	78
Social form	0.656 *	0.011	1
Larger nest size	−0.131	0.896	78
Smaller nest size	2.741 **	0.008	78
Average nest size	0.839	0.404	78
Soil type	16.000 **	<0.001	1
Vegetation cover	19.630 **	<0.001	2

Note: Chi-square tests were performed for social form, soil type and vegetation state, Mann–Whitney *U* test for nest distance, and *t*-test for larger nest size, smaller nest size and average nest size. Asterisks (*) and (**) indicate significance at the 0.05 and 0.01 levels, respectively (two-tailed).

## Data Availability

The original contributions presented in this study are included in the article. Further inquiries can be directed to the corresponding author.
